# Pre-operative anemia and peri-operative transfusion are associated with poor oncologic outcomes in cancers of the esophagus: potential impact of patient blood management on cancer outcomes

**DOI:** 10.1186/s12885-023-10579-x

**Published:** 2023-01-28

**Authors:** Joseph P. Connor, Eric Destrampe, Daniel Robbins, Aaron S. Hess, Daniel McCarthy, James Maloney

**Affiliations:** 1 Department of Pathology and Laboratory Medicine, Section of Transfusion Medicine, University of Wisconsin, 3147 MFCB 1685 Highland Ave, Madison, WI 53705 USA; 2 Department of Pathology and Laboratory Medicine, University of Wisconsin, Madison, WI USA; 3 Department of Anesthesiology, University of Wisconsin, Madison, WI USA; 4 Department of Surgery, Section of Thoracic Surgery, University of Wisconsin, Madison, WI USA

**Keywords:** Anemia, Transfusion, Esophagectomy, Patient blood management

## Abstract

**Background:**

Both Red Blood Cell (RBC) transfusion and anemia are thought to negatively impact cancer survival. These effects have been reported with mixed findings in cancer of the esophagus. The potential impact of the application of restrictive transfusion strategies on this patient population has not been defined.

**Materials and Methods:**

We conducted a retrospective study of esophagectomies and studied cases based on whether they were anemic or were transfused peri-operatively. Clinical characteristics and known clinicopathologic prognosticators were compared between these groups. Survival was compared by Cox proportional hazard modeling. Post-operative transfusions were assessed for compliance with restrictive transfusion thresholds.

**Results:**

Three-hundred ninety-nine esophagectomy cases were reviewed and after exclusions 348 cases were analyzed. The median length of follow-up was 33 months (range 1–152 months). Sixty-four percent of patients were anemic pre-operatively and 22% were transfused. Transfusion and anemia were closely related to each other. Microcytic anemia was uncommon but was evaluated and treated in only 50% of cases. Most anemic patients had normocytic RBC parameters. Transfusion but not anemia was associated with a protracted/prolonged post-operative stay. Transfusion and anemia were both associated with reduced survival however only anemia was associated with decreased survival in multi-variable modeling. Sixty-eight percent of patients were transfused post-operatively and 11% were compliant with the restrictive threshold of 7 g/dL.

**Conclusions:**

Pre-operative anemia and transfusion are closely associated, however only anemia was found to compromise survival in our esophageal cancer cohort, supporting the need for more aggressive evaluation and treatment of anemia. Adherence to restrictive transfusion guidelines offers an opportunity to reduce transfusion rates which may also improve short-term outcomes.

## Introduction

The immune modulating effects of allogeneic red blood cell (RBC) transfusion have been of interest since it was shown in the 1970’s that peri-operative transfusions were associated with improved graft survival in solid organ transplants [1, 2]. These immunosuppressive effects of allogeneic blood transfusions have since been labelled as transfusion related immune modulation or TRIM. Although TRIM still remains poorly understood 50 years later, its immunosuppressive effects are thought to result in poor oncologic outcomes in patients with a variety of solid tumors including malignancies of the esophagus [3–5].

Cancer patients are often anemic and are subsequently transfused RBCs for a number of indications including nutritional deficiencies such as true iron deficiency anemia (IDA), chronic blood loss from friable tumor beds, functional iron deficiency (FID) secondary to cancer induced inflammation, marrow suppressive therapies (including both chemotherapy and radiation), and as a result of acute blood loss associated with surgical management of malignancy (often times after having already been treated with chemotherapy and/or radiation therapy) [6, 7].

The use of pre-surgical neoadjuvant chemo-radiation (NARx) in the management of advanced stage carcinoma of the esophagus has become commonplace. The increasing use of NARx has led to an increase in the incidence of anemia in patients presenting for surgery which in turn increases the use of peri-operative allogeneic RBC transfusion [8, 9]. The effect of anemia and peri-operative RBC transfusions at the time of esophagectomy for cancer have been studied with varying conclusions, although in most studies both transfusion and anemia have had some degree of detrimental effect on cancer survival. Given these data, strategies to reduce the use of peri-operative transfusion in this patient population may not only reduce patient’s exposure to allogeneic blood and the risks of transfusion (transfusion reactions, RBC alloimmunization, and exposure to transfusion transmitted infections) but may also result in improved cancer outcomes by reducing the effects/ risk of TRIM.

Patient Blood Management (PBM) is the process of applying evidence-based guidelines to minimize blood loss and to foster the appropriate use of blood products in order to produce optimal patient outcomes [10–13]. Current recommendations in PBM dictate the use of more conservative transfusion thresholds with hemoglobin levels of 7–8 g/dL being recommended in most clinical settings. A large body of literature now supports these more restrictive strategies, and their implementation has resulted in a significant reduction of blood use [11, 13–18]. More recently, the oncology literature also favors following more restrictive transfusion guidelines, however data in surgically managed cases is limited. In advanced epithelial ovarian cancer, our group has shown that application of restrictive transfusion practice in the peri-operative period can reduce post-operative transfusions by as much as 40 to 90% for thresholds of 8 g/dL or 7 g/dL respectively. This study demonstrated that the application of restrictive transfusion thresholds in cancer surgery can reduce transfusion rates which, based on the negative effects of TRIM, can theoretically contribute to improved cancer outcomes [19].

To determine the effects of anemia and peri-operative transfusions on survival we performed a retrospective study of anemia (both at the time of diagnosis and in the pre-operative setting) and its associated use of peri-operative transfusion over an 11-year period in patients undergoing both primary esophagectomy and those treated with NARx followed by surgery for invasive cancer. We describe the clinical parameters that were associated with both anemia and the use of RBC transfusion and also explore how the application of restrictive transfusion practices could potentially modify the use of transfusion in this patient population.

## Materials and methods

We conducted a retrospective review of all esophagectomies performed at the University of Wisconsin Hospital between September 2005 and July 2016 that were enrolled in a prospective data collection program approved by the University of Wisconsin Heath Sciences Institutional Review Board (IR# 2015–0266) with a waiver for informed consent for this retrospective study. This time frame was selected as it allows a minimum of 5 years post-operative follow-up to assess survival. This retrospective chart review study was done in accordance with all relevant guidelines and regulations. Detailed transfusion data were abstracted from the electronic medical records and added to the ongoing approved database. Data utilized from or added to the existing database for each subject included the date of diagnosis, date of surgery, age at time of diagnosis, gender, Hb and mean corpuscular volume (MCV) at time of diagnosis, treatment with pre-operative NARx, transfusions during NARx, Hb and MCV pre-operatively (which is the same as Hb and MCV at diagnosis in cases not treated with NARx), estimated surgical blood loss, transfusions during surgery (including the number of units transfused and the intraoperative Hb nadir), transfusions in the post-operative period (including the date/post-op day of transfusion, pre- and post-transfusion Hb levels and the number of units transfused).

Anemia was defined using the criteria of the American Society of Hematology as Hb < 12.0 g/dL in women and Hb < 13.5 g/dL in men (https://www.hematology.org/education/clinicians/guidelines-and-quality-care/clinical-practice-guidelines and https://www.hematology.org/education/patients/anemia). Cancer-specific data included pre-operative clinical stage (TNM), histology, surgical/pathologic stage (TNM), date of first recurrence, date of last follow-up, and cancer status at last follow-up as defined by No Evidence of Disease (NED), Alive with Disease (AWD), Died of Disease (DOD), or Died of Other Causes (DOC). Progression-free survival was defined from diagnosis (start of therapy) until the time of first recurrence or last follow-up if there was no recurrence. Overall survival was defined as date of diagnosis until death or date of last follow-up visit for those still alive.

Statistical Analysis: After data collection and exclusions were complete, the entire cohort was divided into groups for analysis based on peri-operative transfusion status (defined as intra-operative and/or post-operative during the primary surgical admission) and pre-operative anemia status. General patient demographics, surgical details, and tumor characteristics were compared between groups by Student’s t-test or Mann–Whitney test and reported as mean ± standard deviation (for continuous variables, based on evaluation of normal distribution by Shapiro–Wilk normality testing) and Pearson’s chi-square or Fisher’s exact test (for categorical variables).

Progression-free and overall survival for individual variables were analyzed using the Kaplan–Meier methodology and compared using the log-rank test. For all univariate tests, we corrected for multiple comparisons by the Benjamini–Hochberg procedure, using a False Discovery Rate (FDR) of 0.05 [20–23]. Variables assessed included anemia at diagnosis, use of neoadjuvant chemotherapy, peri-operative transfusion, positive lymph nodes at surgery, clinical tumor stage, and pathological tumor stage. Two multivariable Cox proportional hazards models were then constructed, one of progression-free survival and one of overall survival. All variables associated with survival a *p* < 0.1 in univariate tests were included in initial multivariable models. Variables with a Type III Wald test *p* < 0.05 were retained in the final model. Variables included in the two final models were then verified by repetition using forward-selection.

Statistical analyses were performed using Graph Pad Prism software (GraphPad Software, Inc La Jolla, CA) and SPSS (IBM Corp. Armonk, NY) programs.

## Results

### Study population demographics

Between September 2005 and July 2016, 399 patients underwent esophagectomy with malignancy being the most common indication. Fifty-one cases were excluded from analysis: 26 with pre-invasive disease only, 11 esophagectomies that were done for achalasia, stricture, or trauma without cancer, 9 surgeries that were done as salvage of recurrent cancer, and 5 cases with an additional primary cancer at the time of esophagectomy. After these exclusions there were 348 cases of esophagectomy for analysis.

Consistent with the known demographics of patients with esophageal cancers our study population was on average 65 ± 10 years old, predominantly male (79%), with clinical stage III disease (73%) and adenocarcinoma histology (85%). Two thirds of the patients were treated with NARx. The median follow-up time for study subjects was 33 months (time to death or last follow-up encounter) with a range of 1–152 months. Anemia was found in 42% of cases at diagnosis and the rate of anemia increased to 64% at the time of surgery with a net drop in Hb between diagnosis and surgery of -1.2 + 2.0 g/dL for those treated with NARx. For the entire population, the peri-operative transfusion rate was 22% which is lower than the historically reported transfusion rates for this population [24–27]. Twenty-two patients were transfused intra-operatively with a mean of 1.9 ± 1.1 units given and an intra-operative Hb nadir of 8.1 ± 0.8 g/dL. All patients transfused intra-operatively were anemic going into surgery. Sixty-eight cases were transfused in the post-operative period, including 12 of the 22 patients who were already transfused during surgery. These 68 cases, which are described in more detail below, were transfused a mean of 1.5 ± 0.6 units for a mean pre-transfusion Hb of 8.1 ± 0.9 g/dL.

### Analysis based on transfusion status

Patient’s demographics and cancer treatment are outlined in Table 1 according to peri-operative transfusion status. Patients who were transfused were more often anemic, both at the time of diagnosis and pre-operatively, had more surgical blood loss, lower intra-operative Hb values, and longer hospitalizations indicative of more complex peri-operative clinical course. The original pre-operative clinical stage distribution and tumor histology were similar between patients that were transfused and those not transfused, and a similar proportion of patients in each group were treated with NARx. For those treated with NARx, transfused patients were less likely to have achieved a complete pathologic response (no grossly visible tumor at the time of surgery, pathologic stage T0, 17% vs 26%) indicating that the presence of residual tumor after NARx potentially resulted in more pre-op anemia and or more technically complicated/difficult surgeries resulting in an increased need for transfusion.

**Table 1:** Patient’s Demographics and Cancer Treatment Characteristics Based on Transfusion Status

	Transfused (*n* = 78)	Not Transfused (*n* = 270)	*p*-value
Age *	67 ± 9 years	65 ± 9 years	0.09
Gender	12 (15%) Female	62 (23%) Female	0.16
	66 (85%) Male	208 (77%) Male	
Anemic at Diagnosis** 120/289 (42%)	41 (61%)	79 (36%)	0.0002
Clinical T Stage	1 8 (12%) 2 7 (10%) 3 51 (75%) 4 2 (3%)	1 31 (14%) 2 32 (14%) 3 164 (72%) 4 0 (0%)	0.26
Tumor Histology	12 (16%) Squamous 65 (84%) Adenoca	37 (11%) Squamous 288 (89%) Adenoca	0.33
Treated with NARx	53 (68%)	163 (60%)	0.70
Diagnosis to OR (days)*^,$^	130 ± 2 days	136 ± 39 days	0.39
Complete Response (CR) to NARx^$^	12 (23%)	66 (40%)	*P* = 0.021
Anemic Pre-operative** 216/338 (64%)	67 (86%)	149 (57%)	< 0.0001
Estimated Blood Loss (EBL)*	383 ± 274 ml	257 ± 166 ml	< 0.0001
Pathologic/Surgical T Stage	0 13 (17%) 1 15 (19%) 2 17 (22%) 3 30 (38%) 4 2 (3%) In-Situ 1 (1%)	0 69 (26%) 1 70 (26%) 2 44 (16%) 3 83 (31%) 4 0 (0%) In-Situ 3 (1%)	*P* = 0.032
Lymph Node Status	50 (64%) Negative	172 (64%) Negative	1.00
	28 (36%) Positive	97 (36%) Positive	
Hospital Length of Stay *	19 ± 20 days	9 ± 4 days	< 0.0001

^*^ Mean + S.D. reported

^**^ anemia diagnosed as Hb < 12.0 g/dL for women and Hb < 13.5 g/dL for men

^$^for cases treated with neoadjuvant chemo/radiotherapy only

### Analysis based on pre-operative anemia status

**Fig. 1:**
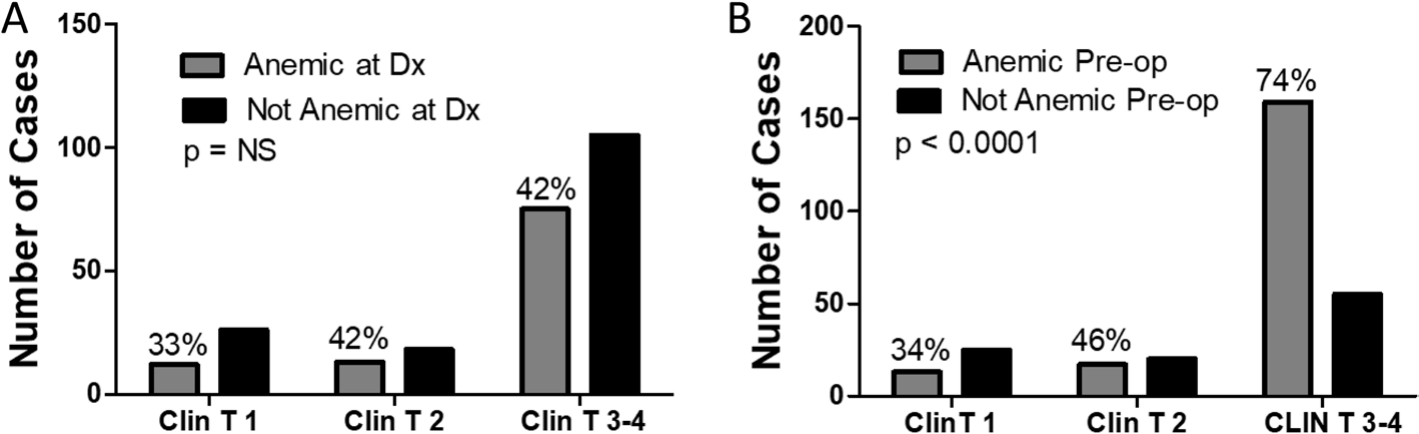
Chi Square analysis demonstrates that anemia was equally distributed across clinical stages of disease at diagnosis but was far more likely to be present pre-operatively in patients with clinical stage III disease, who were e likely to be treated with neo-adjuvant therapy

Table 2 shows the patient’s demographics and cancer treatment between patients that went to surgery anemic and those that were not anemic. Anemic patients were 3–4 years older, more often male, and were anemic at diagnosis in over 60% of cases. Pre-operative anemia was more often seen in clinical stage III disease and therefore was also more commonly associated with the use of NARx (77% of anemic patients received NARx). Unlike the pre-operative setting, anemia was equally distributed across clinical T stages at diagnosis (Fig. 1). Of the patients that were anemic at the time of surgery, 69 of them were not anemic when they initially presented for evaluation and treatment. All but 2 of these cases were clinical stage III and all of them were treated with NARx. In patients treated with NARx, pre-operative anemia was also associated with a long interval between diagnosis and surgery, however the rate of complete clinical response to NARx was similar between anemic and not anemic cases. There was no difference in surgical blood loss between the groups, however, residual disease was more often found at the time of surgery with more pathologic/surgical T (yPT III) stage III cases in the anemic group. The post-operative length of stay was not different between the groups which, when coupled with similar volumes of surgical blood loss, would suggest that pre-operative anemia was not associated with more complex or anatomically challenging surgery as was seen in the analysis for transfusions.

**Table 2:** Patient’s Demographics and Cancer Treatment Characteristics Based on Pre-operative Anemia Status

	Anemic (*n* = 216)	Not Anemic (*n* = 122)	*p*-value
Age*	68 ± 10 years	64 ± 10 years	0.001
Gender	37 (17%) Female	35 (29%) Female	0.018
	179 (83%) Male	87 (71%) Male	
Anemic at Diagnosis** 120/289 (42%)	113 (62%)	7 (7%)	0.0002
Clinical T Stage (*N* = 289)	1 13(7%) 2 17 (9%) 3 157 (83%) 4 2 (1%)	1 25 (25%) 2 20 (20%) 3 55 (55%) 4 0 (0%)	< 0.0001
Tumor Histology	33 (16%) Squamous 178 (84%) Adenoca	15 (12%) Squamous 106 (88%) Adenoca	0.52
Treated with NARx	166 (77%)	47 (39%)	< 0.0001
Diagnosis to OR (days)*^,$^	148 ± 539 days	130 ± 39 days	0.01
Complete Response (CR) to NARx^$^	57 (34%)	19 (40%)	0.49
Estimated Blood Loss (EBL)*	280 ± 180 ml	289 ± 215 ml	0.72
Pathologic/Surgical T Stage	0 59 (27%) 1 36 (17%) 2 42 (20%) 0 75 (36%) 1 2 (1%) In-Situ 1 (< 1%)	0 21 (17%) 1 46 (38%) 2 18 (15%) 0 34 (28%) 1 0 (0%) In-Situ 3 (2%)	0.0002
Lymph Node Status	138 (64%) Negative	78 (64%) Negative	1.00
	77 (36%) Positive	44 (36%) Positive	
Hospital Length of Stay *	11 ± 11 days	11 ± 11 days	0.66

^*^ Mean + S.D. reported

^**^ anemia diagnosed as Hb < 12.0 g/dL for women and Hb < 13.5 g/dL for men

^$^ for cases treated with neoadjuvant chemo/radiotherapy only

### Univariable and multivariable survival analysis

**Fig. 2:**
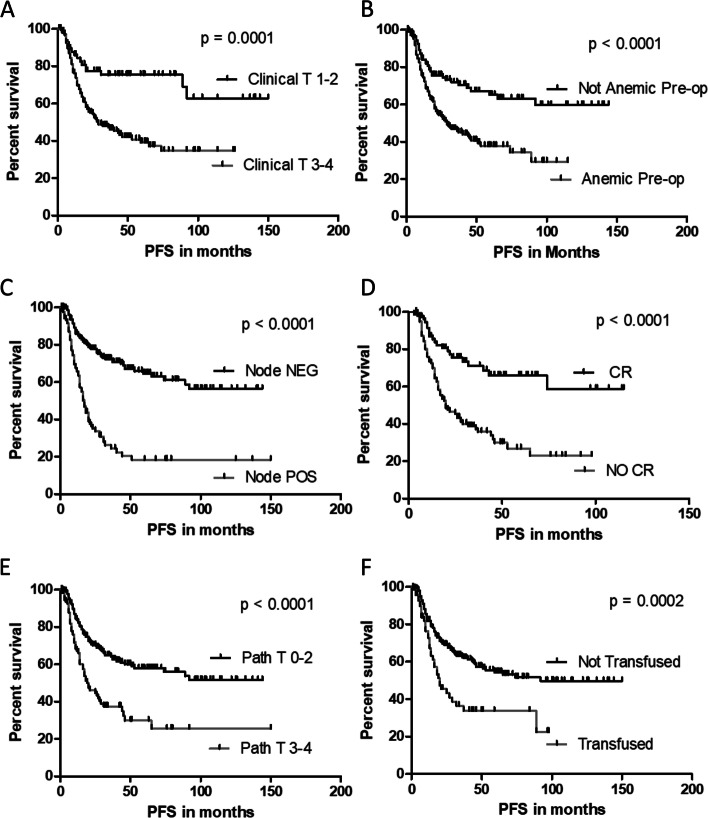
Differences in progression free survival were compared with the log-rank test with significance set at *p* ≤ 0.05. All *p*-values remained significant after correction by the Benjamini–Hochberg procedure with an FDR of 0.05. Of the clinical parameters described in Tables 1 and 2, both pre-operative anemia and peri-operative transfusion were associated with reduced progression free survival. All associations with a *p* < 0.1 were included in multivariable models as described in Materials and Methods and Results

**Fig. 3:**
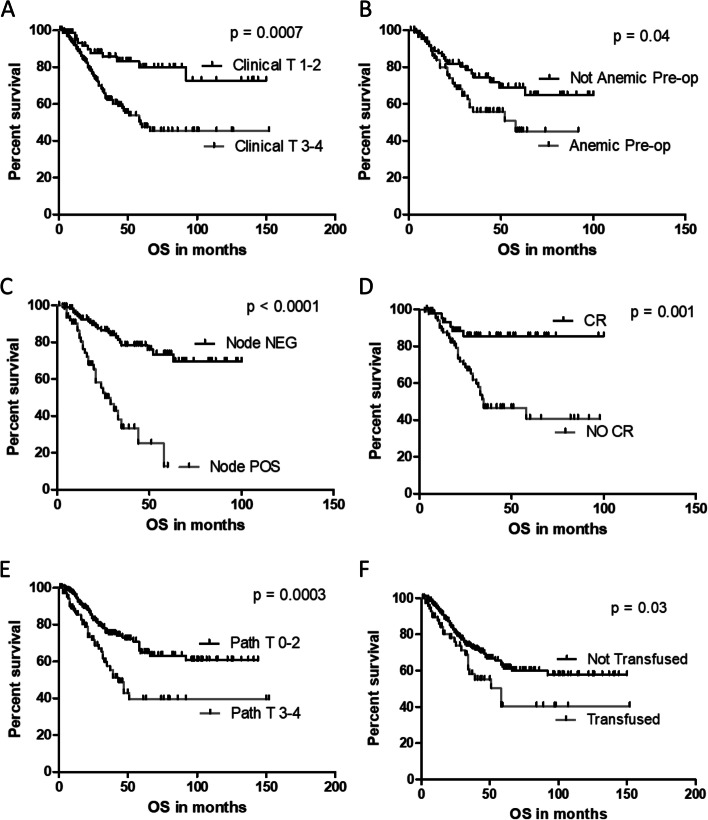
Differences in overall survival was compared with the log-rank test with significance set at *p* ≤ 0.05. All *p*-values remained significant after correction by the Benjamini–Hochberg procedure with an FDR of 0.05. As was seen for progression free survival, both pre-operative anemia and peri-operative transfusion were associated with reduced overall survival. All associations with a *p* < 0.1 were included in multivariable models as described in Materials and Methods and Results

Of the clinical parameters described in Tables 1 and 2, six of them were predictive of both progression free and overall survival in univariate analysis and all 6 remained significant after applying a Benjamini–Hochberg procedure FDR of 0.05 (Figs. 2 and 3 respectively). Both pre-operative anemia and peri-operative transfusion were associated with poor progression-free survival (Fig. 2, B and F) and worse overall survival (Fig. 3, B and F). In multivariable Cox Proportional Hazard models, progression free survival was significantly associated with the use of NARx (hazard ratio [HR]: 0.61, 95% confidence interval [95%CI]: 0.37 – 0.99, *p* = 0.047) and inversely associated with peri-operative transfusion (HR: 1.69, 95%CI 1.10 – 2.58, *p* = 0.016), anemia at time of surgery (HR: 1.92, 95%CI: 1.18 – 3.12, *p* = 0.009), positive nodal status (HR: 3.22, 95%CI 2.19 – 4.73, *p* < 0.001), and increasing clinical stage (HR for stage IV vs I: 23.22, 95%CI: 2.53 – 212.82, overall *p* < 0.001). Similarly, overall survival was significantly associated with the use of NARx (HR for mortality: 0.50, 95%CI: 0.36 – 0.69, *p* = 0.031) whereas the hazard of death was higher among patients with anemia at time of surgery (HR: 2.56, 95%CI: 1.37 – 4.59, *p* = 0.003), positive lymph nodes at surgery (HR: 2.58, 95%CI 1.58 – 4.20, *p* < 0.001), increasing clinical stage (HR for stage IV vs I: 27.89, 95%CI: 3.19 – 244.19, overall *p* = 0.002) and increasing pathological stage (HR for stage V vs 0: 20.75, 95% CI: 4.28 – 100.58, *p* = 0.009). Transfusion was not a significant, independent predictor of overall survival in this model.

### Anemia evaluation and treatment

**Fig. 4:**
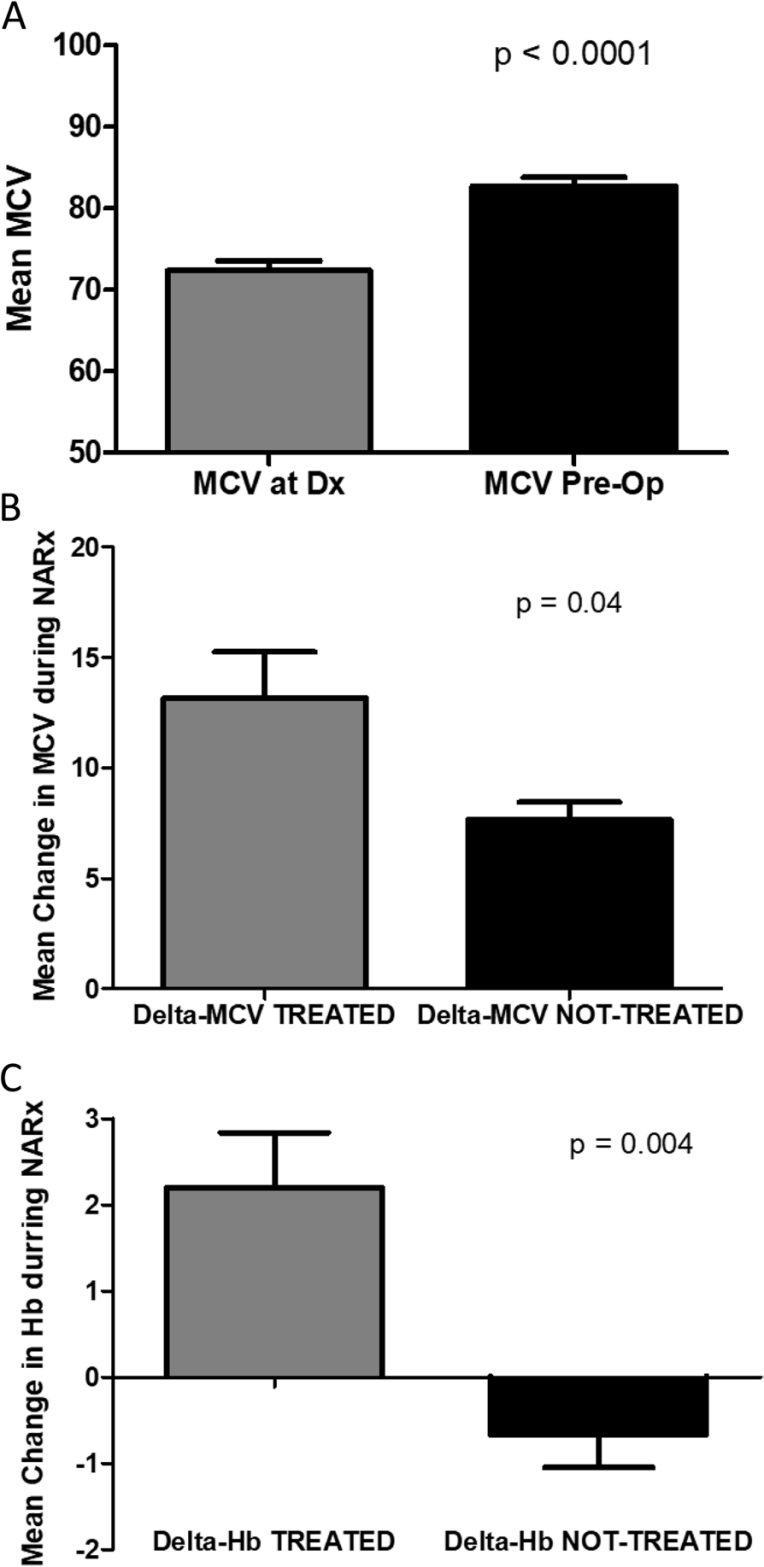
Changes in Mean Corpuscular Volume (MCV) and Hemoglobin (Hb) values during NARx. MCV for the whole group increased during treatment (**A**) with significantly more increase in the subjects who had treatment for iron deficiency (**B**). Similarly, treated iron deficiency resulted in a net gain of Hb compared to further loss of Hb in untreated patients (**C**)

Of 348 cases studied, an MCV value at the time of diagnosis was available in 242 (70%) with a mean value of 89.0 ± 6.5 fL (normal range 80–100 fL). Seventeen of these 242 cases were frankly microcytic with MCV < 80fL and 16 of these 17 were anemic. Of the 17 microcytic cases, 13 were treated with NARx and 8 of them (47%) had iron studies, all of which confirmed iron deficiency. Of the 8 documented iron-deficient cases 4 of them were treated for their anemia during NARx; two were treated with iron supplementation, one with transfusion and one with both transfusion and iron therapy. Another three cases with microcytic MCV values were anemic and transfused during NARx without assessment of their iron status giving a total of 7 treated microcytic anemia cases and 6 microcytic anemias that did not have iron evaluations or any anemia treatment during NARx. Of the 7 treated cases, 3 of them were transfused peri-operatively; two were transfused intra-operatively with blood loss of over 500 ml and the third was transfused one RBC unit in the post-operative period. Pre-operative MCV was available in all 13 patients that were microcytic at diagnosis and, as a group, there was an increase in MCV during NARx with a mean improvement of 10.2 ± 3.6 fL (range 4–19 fL, Fig. 4A). The seven cases that were treated with either transfusion or iron therapy had near twice the increase in MCV at 13 fL compared to 7 fL in those that were not treated (*p* = 0.04 by Student’s t-test, Fig. 4B). The 7 treated cases also had a significant increase in mean Hb levels during NARx of 2.2 ± 1.6 g/dL compared to a loss of Hb of -0.7 ± 0.9 g/dL in the 6 non-treated cases (*p* = 0.004 by Student’s t-test, Fig. 4C). For the 3 patients that were found to be iron deficient and were treated with iron supplementation, the mean change in Hb and MCV were 3.6 g/dL and 16.3 fL respectively. The cancer mortality for the 13 microcytic patients was higher than the entire cohort at 54% vs 37% died of cancer at the time of analysis; however, due to the very small number of cases with complete iron work-up and treatment, a full survival analysis for treated iron deficiency vs not treated cases was not done. In addition to the microcytic cases described, another 43 patients were at risk of becoming iron deficient during NARx given the presence of low-normal MCV (80- 90fL) at diagnosis. A total of 217 patients had an MCV in the normocytic range (80–100 fL) and of these cases 83 (38%) were anemic, potentially representing early iron deficiency or functional iron deficiency. The normocytic anemia cases were diffusely distributed across MCV values; however, iron status evaluations were rare, making the incidence of iron deficiency or functional iron deficiency in this group impossible to define. An additional 8 patients were macrocytic with MCV values over 100 fL at diagnosis. Only 1 of these cases was anemic and, none of them had evaluation of vitamin B12 or folate before starting NARx. At the time of surgery an additional 12 cases had MCV > 100 fL and 75% of these patients were anemic, again, without B12 and /or folate assessment.

### Compliance with restrictive transfusion guidelines

In the post-operative period, 68 patients received 104 transfusions; 42 patients had a single post-operative transfusion, 18 had 2, 6 patients had 3 and 2 patients had 4 post-operative transfusion events. All of these transfusions were ordered routine and none of them were associated with clinically identifiable active bleeding or re-operation. The mean pre-transfusion Hb level was 8.1 + 0.9 g/dL (range 7.6–10.9 g/dL) and only 11 (11%) had a pre-transfusion Hb of < 7 g/dL, the most common, evidence-based, and restrictive transfusion threshold supported by effective PBM. In other words, as many as 89% of post-operative transfusions could be considered over-transfusion and potentially unnecessary if the 7 g/dL threshold were in place. The percent of compliant transfusions increases to 41% if the more liberal threshold of 8 g/dL were to have been applied. Consistent with current recommendations and effective PBM strategies, many of the post-operative transfusions (59%) were single unit transfusion events.

## Discussion

In our retrospective study of 348 esophagectomies for malignancy, we verify that anemia is prevalent in patients with cancer of the esophagus and that without assessment and treatment, the use of NARx increases the incidence of anemia at the time point of surgery. The analysis of the retrospective data suggests that both pre-operative anemia and the use of peri-operative transfusion were both related to poor oncology outcomes. The literature in this area documents, and we verify here, that anemia and transfusion are very strongly associated with each other, making it difficult to separate the influence of each from the other even when multivariable analysis is applied. Since both the active management of pre-operative anemia and the reduction of unnecessary transfusions are components of effective PBM, [13, 14, 28–30] our study demonstrates that both of these areas offer opportunities for improvement in the management of patients with carcinoma of the esophagus, especially when treating with NARx.

The data we present underscore the rationale behind the development of institutional PBM programs that can both educate providers and facilitate improvement in patient care. Such programs should include the routine and consistent evaluation of anemia, an assessment of the risk of developing anemia, as well as, the continued reduction of transfusions that can contribute to unfavorable patient outcomes [12–14]. Based on the data presented here and from several other studies [17, 19, 31] we are actively developing a robust, institution wide PBM program designed to improve both pre-operative anemia evaluation and management and to improve compliance with restrictive transfusion care in all of our clinical services [17, 31]. Our PBM program has implemented an electronic decision support tool built into our electronic medical record to promote compliance with restrictive transfusion thresholds, and we have piloted a data-driven provider education program to reinforce restrictive transfusion and to promote the preferred use of single unit transfusion. Another factor complicating modern oncology care is that many patients receive various aspects of their treatment at a variety of facilities making coordination of care a realistic obstacle to the uniformity of care. This is especially problematic in terms of the consistent and standard approaches to anemia both before and during neo-adjuvant therapies as these treatments are increasingly being provided at institutions other than where the planned surgical procedures are being performed.

The complex problem of anemia in patients with cancer is well described in the clinical literature [6]. In a review paper by Gaspar in 2015, it was noted that 40 to 60% of cancer patients are anemic at some point during their initial evaluation. This anemia was most often associated with chronic, cancer associated inflammation and the resulting hepcidin-based functional iron deficiency. Other nutritional-related anemias including microcytic, true iron deficiency and B12/folate related megaloblastic anemias were also prevalent, demonstrating the diversity of the problem of anemia in this patient population. [7] Underscoring the complexity of the problem Gillespie and colleagues remind us that anemia often may develop slowly in cancer patients and that many patients are functionally compromised before their hemoglobin value drops to an abnormal range making it difficult to aggressively treat the anemia early [32]. In addition, the all-too-common nutritional deficiency associated with poor caloric intake and the tumors’ parasitic use of available calories present additional challenges in addressing anemia in these patients [33–35].

Functional iron deficiency (FID) is defined as the presence of low circulating iron levels, low Transferrin Saturation (< 20%) and a normal or increased serum ferritin in the setting of systemic inflammation [36, 37]. Evidence of inflammation is most commonly demonstrated through the elevation of the inflammatory marker C-reactive protein (CRP). Historically, this has been termed anemia of chronic disease or anemia of chronic inflammation. In the presence of inflammation, elevated hepcidin levels prevent appropriate mobilization of normal iron stores for effective erythropoiesis [38, 39].

When anemia is present in a patient with advanced cancer, it is estimated that at least 60–70% of cases are due to the presence of FID and, as we verify in the present study, present with normocytic RBC indices [40]. FID can be present before clinical anemia is evident, and this may be one of the reasons for the increasing incidence of anemia after completion of NARx as seen in our study population.

FID has been shown to respond well to intravenous iron therapy in randomized clinical trials; however, its diagnosis requires a more detailed laboratory assessment [41–43]. In addition to the standard IDA lab testing (iron levels and ferritin), transferrin saturation and CRP (or another marker of inflammation) are minimal requirements. Some newer and investigational tests such as hepcidin level and reticulocyte Hb values may also be of value in these algorithms. As highlighted in the current study, basic assessments of anemia or risk of anemia are only performed in a small fraction of patients emphasizing the need to make a detailed anemia assessment in all cancer patients (including assessments for FID) routine practice, especially for those patients with planned NARx. Implementation of routine anemia evaluation will improve our ability to identify patients with FID who may benefit from intravenous iron therapy even before definitive anemia is detected. Such a standard process could facilitate early intervention and thus negate some of the negative impact of NARx on the progression of anemia in these patients.

Focusing on the adequate assessment of iron status with the potential increased opportunity for aggressive management of both IDA and FID is a key component of our institution’s evolving PBM program. The creation of common clinical protocols for the assessment of iron status (or other anemia related assessments such as B-12 and folate levels), its interpretation, and referral guidelines for therapy when indicated is an evolving approach.

Pre-operative anemia has been extensively studied in a number of solid malignancies, most notably in cancer of the lower GI tract/colon [44, 45]. Pro-active identification, assessment, and treatment of anemia, especially iron deficiency, has been shown to reduce the use of allogeneic transfusion, reduce the incidence of post-operative complications, and improve the overall post-operative recovery process [45–47].

Previous studies have noted an association between anemia or transfusion and worse oncologic outcomes in treatment of esophageal cancer, but anemia and transfusion have rarely been studied together, and the effect of preoperative anemia management has not been examined. There is some variation in anemia- and transfusion-associated outcomes depending on how the cancer is treated. In squamous cell esophageal cancer treated with primary radiotherapy alone, pre- treatment anemia is associated with decreased overall survival [27]. In patients treated with surgery alone, transfusion is associated with recurrence [48] and mortality [49]. [48] [25] Among patients treated with NARx and esophagectomy, transfusion is associated with excess mortality [24], and patients with anemia are more likely to need transfusion [26], and to develop surgical site infections [26].

Although our statistical modeling using this large retrospective database (and that found in the literature cited) shows that anemia can be an independent predictor of cancer outcomes, it is also known that anemia is directly related to more advanced disease and/or more aggressive tumor biology and is a direct indication to proceed to transfusion. Therefore, it is reasonable to assume that neither anemia nor transfusion can be completely separated from tumor biology and/or extent of disease in terms of prognosis. That being said, the assessment and treatment of anemia and the optimal use of RBC transfusion are both variables that can be modified in an active attempt to improve patient outcomes. Until the time comes when we can more specifically or directly modify tumor biology, it stands to reason that we can and should intervene when and where we can to maximize the possibility of improving outcomes [50, 51]. In this way, the active management of anemia and reducing unindicated transfusion can be looked at similarly to the concept of active management of other patient co-morbidities such as hypertension or diabetes where optimal management of these non-cancer, medical issues can allow for optimal application of cancer therapy and thus increase the chances of improved outcome [52, 53]. Although anemia has been consistently shown to be associated with increased morbidity and mortality whether or not the treatment of anemia in the pre-operative setting can result in improved post-operative patient outcomes, especially cancer survival, remains elusive [54] and the optimal role of and protocols for pre-operative anemia treatment remain unknown. Several randomized clinical trials (RCTs) have attempted to address this question with none of them focusing specifically on patients with cancer of the esophagus [55, 56]. One frequently referenced RCTs by Richards and colleagues attempting to answer this question showed no benefit of pre-op iron treatment [55]. However this study included a broad variety of surgery and patient types and its primary outcomes of need for transfusion and 30 day mortality were likely too narrow to identify other potential benefits such as reduced post-operative infections [57]. Given the diversity of patients included studies like this are most likely underpowered to account for the variety of cofounding factors that could affect the study’s narrow endpoints. Given the limitations of the trials done to date, either very large and more completely controlled trials or disease specific trials may be the best options for addressing the question of clinical benefit [57–60].

Finally, in a 2018 study of over 7000 cases of esophageal carcinomas at 182 separate clinical sites, Towe et al. provided one of the few studies to evaluate the effects of restrictive transfusion thresholds and modern PBM strategies in the surgical management of esophageal cancer [61]. They noted a significant improvement in transfusion rates when restrictive guidelines were imposed bringing the rate to 23%, which is similar to the overall transfusion rate in the current study. It was also noted that the fewer transfusions resulted in significantly lower morbidity for the surgical population in general. As our group has reported in the surgical management of advanced ovarian cancer, adherence to more restrictive transfusion thresholds can result in marked reduction in the number of transfusions, especially in the post-operative period. Our current study verifies and expands on these results with the observation that, even with appropriately restrictive transfusion thresholds, attention to the evaluation and treatment of anemia at both time of diagnosis and in the pre-operative setting is needed in this patient population. We hypothesize that this is an opportunity for improved outcomes by further decreasing the need for allogeneic transfusions and subsequent reduced morbidity and mortality.

The strengths of our current study include the availability of a larger sample size from a single institution with the surgical care being provided by a limited number of surgeons (4 surgeons for all 399 cases) over the entire study period. In addition, our study population has a high rate of complete data on peri-operative transfusion details (97% complete data) with consistent and long**-**term patient follow-up data. The major weakness of our study is its retrospective nature and the associated lack of complete information about anemia evaluations as there was no standard approach over the study time-period. We also have to acknowledge that during the last years of patient recruitment for this study PBM evolved and taught the use of restrictive transfusion thresholds. If and how PBM influences this study cannot be analyzed retrospectively. Our institution itself implemented PBM after the study recruitment was terminated in 2016, so the influence should be within a low extent.. Additionally, our study suggests that transfusion is associated with increased post-operative morbidity as demonstrated by increased length of hospitalization; however, the available dataset does not include the required post-operative information specific to any individual complications to definitively make this conclusion. Finally, as mentioned already, the demonstration of a statistical correlation does not verify that interventions to address anemia or transfusion will result in improved outcomes and therefore more focused and-or better controlled RCTs are still needed to address the optimal role of pre-operative anemia management in patients with esophageal cancers.

In conclusion this study supports increased efforts to actively manage cancer associated anemia and to reduce the use of allogeneic RBC transfusions in the peri-operative period in patients with malignancies of the esophagus.

## Data Availability

The datasets used and/or analyzed during the current study can be made available from the corresponding author on reasonable request.
